# Protocol to characterize basement membranes during kidney development using mass spectrometry-based label-free quantitative proteomics

**DOI:** 10.1016/j.xpro.2023.102741

**Published:** 2023-11-29

**Authors:** Mychel R.P.T. Morais, Pinyuan Tian, Ronan O’cualain, Craig Lawless, Rachel Lennon

**Affiliations:** 1 Wellcome Centre for Cell-Matrix Research, University of Manchester, Manchester M13 9PT, UK; 2 Department of Paediatric Nephrology, Royal Manchester Children’s Hospital, Manchester University Hospitals NHS Foundation Trust, Manchester M13 9WL, UK

**Keywords:** Metabolism, Proteomics, Mass Spectrometry

## Abstract

Basement membranes are specialized extracellular matrices formed by highly insoluble structural proteins and extracellular matrix (ECM)-bound components that provide structural and signaling support to tissues and are dynamic during development. Here, we present a mass spectrometry-based label-free quantitative proteomics protocol to investigate basement membranes and define their composition using samples from human kidney organoids and mouse fetal kidneys. This protocol facilitates the study of basement membrane and other ECM components during development to improve our understanding of matrix regulation and function.

For complete details on the use and execution of this protocol, please refer to Morais et al.^1^

## Highlights

• A detailed proteomics pipeline to characterize extracellular matrix
• A protocol for elucidating matrix dynamics during development and disease
• Broad application to cell-based systems and organ or tissue analysis
• Workflows for proteomic data integration

## Abstract (editor highlights)

Publisher’s note: Undertaking any experimental protocol requires adherence to local institutional guidelines for laboratory safety and ethics.

## Graphical abstract

**Figure:**
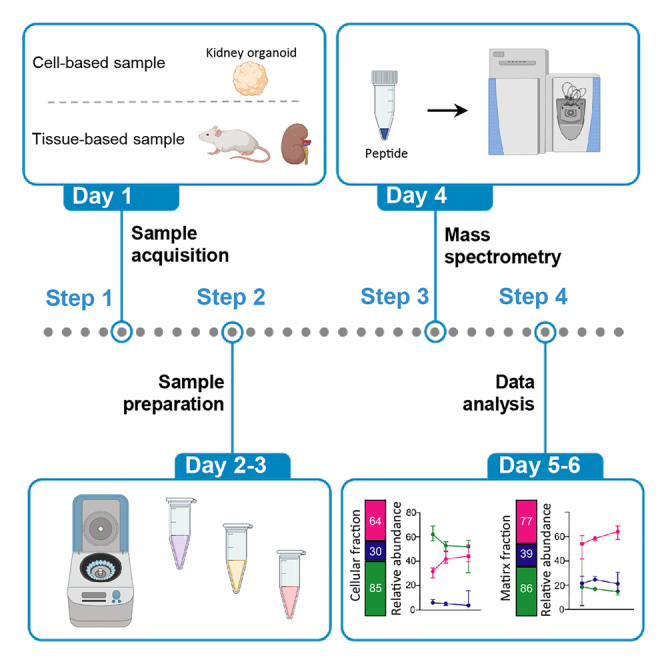

## Before you begin

Basement membranes are specialized extracellular matrices that surround and separate tissue, providing them with a structural and signaling platform that influences tissue shape and function, regulates cellular aspects processes such as polarity and survival via signal transduction through cell surface receptors (e.g., integrins, dystroglycan).^2^^,^^3^^,^^4^^,^^5^ The protocol below describes a streamlined framework to enrich fetal mouse kidneys and human iPSC-derived kidney organoids for basement membrane proteins, as well as other extracellular matrix proteins, through chemical fractionation and further deep proteome profiling with discovery label-free quantitative mass spectrometry.

### Institutional permissions

All mouse experiments described here were performed under the approval of the Animal Ethics Committee of the Institute of Biomedical Sciences (University of São Paulo, protocol 019/2015).

### Experimental design for sample collection for proteome profiling

**Timing: 25 days for kidney organoid differentiation; 19 days for mouse fetal kidney acquisition**

**Figure 1:**
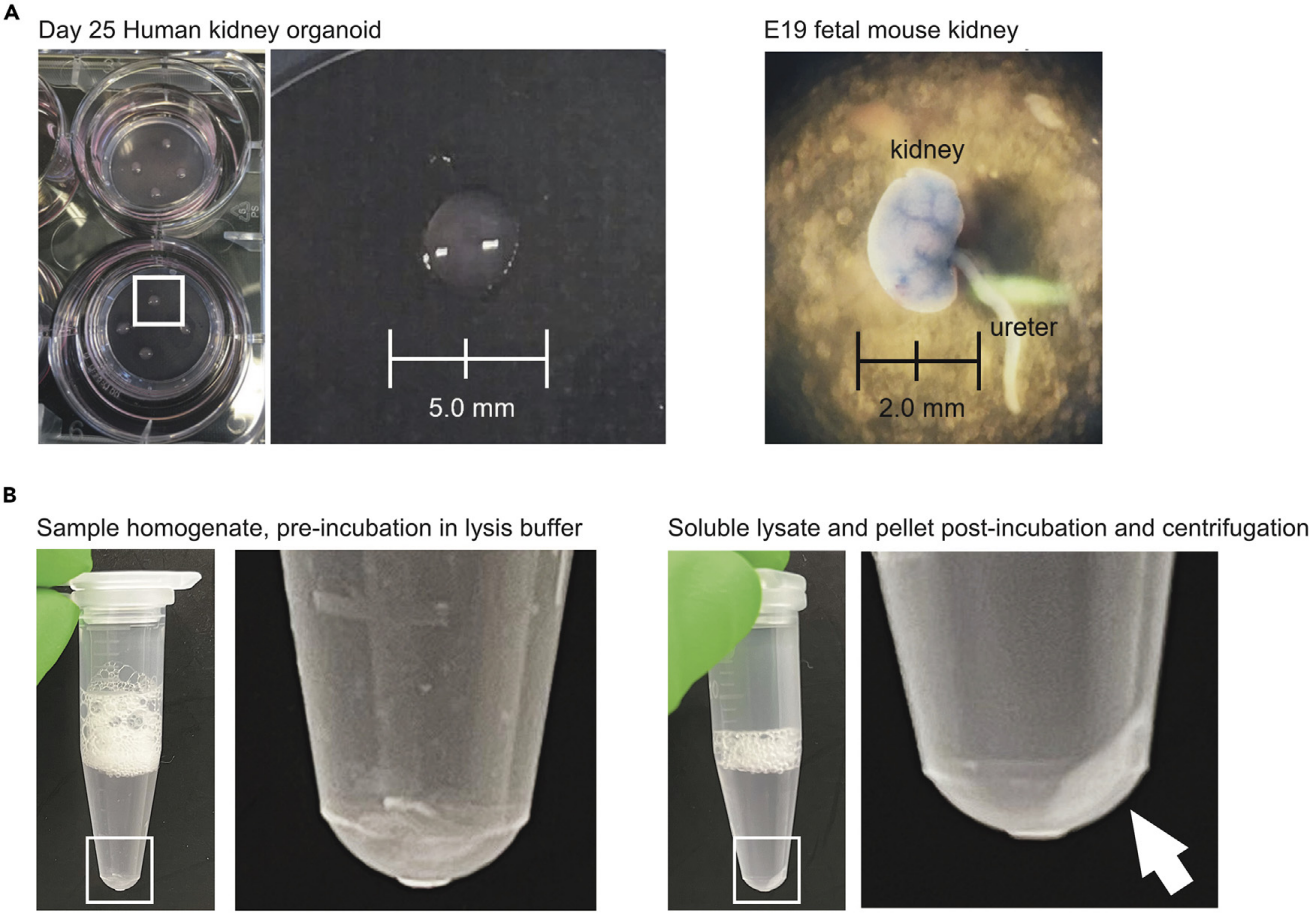
Image of key steps (A) Representative images of day 25 human kidney organoids, and E19 fetal mouse kidney with scale bars, both processed for extracellular matrix enrichment and further proteomic analysis with mass spectrometry. (B) Tissue homogenate (before and after incubation and centrifugation in lysis buffer) during fractionation for enrichment for matrix proteins.

For this protocol, we will describe the procedures done using human kidney organoids and fetal mouse kidneys (Figure 1A).

1. Human kidney organoid culture.
  a. Kidney organoids are generated from human pluripotent stem cells (PSCs).^6^
  b. Collect kidney organoids from inserts on days 14, 18, and 25, wash with PBS, snap-freeze in liquid nitrogen, and store at −80°C until further processing.
2. Mouse mating schedule and sample acquisition.
  a. To obtain fetal mouse kidney samples, breed the mice by mating females and males for 12 h, and check on the next morning for detection of pregnancy, which is confirmed based on visualization of a vaginal plug.
  b. Keep pregnant females until the 19^th^ day of gestation and then anesthetize them with tribromoethanol (0.025 mL/g of body weight) to submit to caesarian surgery for collection of the fetuses.
  c. Dissect fetal mouse kidneys using clean forceps and surgical scissors, snap-freeze in liquid nitrogen, and store at −80°C until further processing.

**CRITICAL:** Discard used blades and/or needles used for dissecting the mouse samples in a biohazard sharps container. Ensure the use of adequate personal protective equipment, including a lab coat, gloves (e.g., latex, nitrile), face mask, and eye protection.

## Key resources table

**Table:** 

REAGENT or RESOURCE	SOURCE	IDENTIFIER
**Chemicals, peptides, and recombinant proteins**		

Acetonitrile (ACN)	Thermo Fisher Scientific	Cat# 047138.M1
Dithiothreitol (DTT)	Fisher Scientific	Cat# BP172-5
EDTA-free Roche complete protease inhibitor cocktail	Sigma-Aldrich	Cat# 4693116001
Formic acid Optima	Fisher Scientific	Cat# A117-50
Iodoacetimide (IAM)	Sigma-Aldrich	Cat# I1149
Methanol (MetOH)	Thermo Fisher Scientific	Cat# A456
Ammonium hydroxide (NH_4_OH)	Sigma-Aldrich	Cat# 338818
Sodium chloride (NaCl)	Sigma-Aldrich	Cat# S9888
OLIGO R3 reversed-phase resin	Applied Biosystems	Cat# 1133903
Phosphate-buffered saline (PBS)	Thermo Fisher Scientific	Cat# D8537
Phosphoric acid	Acros Organics	Cat# 463861000
Pierce water, LC-MS grade	Thermo Fisher Scientific	Cat# 10763295
Sequencing grade modified trypsin, frozen	Promega	Cat# V5111
Sodium dodecyl sulfate (SDS)	Sigma-Aldrich	Cat# 75746
Triethylammonium bicarbonate (TEAB)	Sigma-Aldrich	Cat# T7408
Tris	Fisher Scientific	Cat# 10103203
Triton X-100	Sigma-Aldrich	Cat# X100
Ethylenediaminetetraacetic acid (EDTA)	Fisher Scientific	Cat# 10335460

**Deposited data**		

Mass spectrometry proteomics data	Morais et al.^1^	PRIDE: PXD025838, PXD025874, PXD025911, PXD026002, PXD022219

**Experimental models: Cell lines**		

Cell line (*Homo sapiens*)	Wood et al.^7^	-

**Experimental models: Organisms/strains**		

Strain, strain background (*Mus musculus*)	University of São Paulo (Brazil)	-

**Software and algorithms**		

Proteome Discoverer v.2.3.0.523	Thermo Fisher Scientific	https://www.thermofisher.com/uk/en/home/industrial/mass-spectrometry/liquid-chromatography-mass-spectrometry-lc-ms/lc-ms-software/multi-omics-data-analysis/proteome-discoverer-software.html/
SonoLab software v.8.2	Covaris	https://www.covaris.com/products-services/instruments/sonolab-software/
Cytoscape v.3.8.1	Cytoscape team^8^	https://cytoscape.org/
GraphPad Prism v.9.4.1	Dotmatics	https://www.graphpad.com/
R Environment	The R Project for Statistical Computing^9^	https://www.r-project.org/
RStudio v.1.2.5042	RStudio Team^9^	https://www.rstudio.com/
ggplot2 package v.3.3.2	Wickham et al.^10^	https://ggplot2.tidyverse.org/
ComplexHeatmap package v2.2.0	Gu et al.^11^	(http://www.bioconductor.org/packages/release/bioc/html/ComplexHeatmap.html)

**Other**		

microTUBE AFA fiber pre-slit snap-cap 6 × 16 mm	Covaris	Cat# 520045
microTUBE-500 AFA fiber screw-cap	Covaris	Cat# 520185
S-Trap micro columns	ProtiFi	-
96-well S-Trap plate	ProtiFi	-
Corning FiltrEX 96-well filter plates	Merck	Cat# CLS3511
Abgene 96-well microplates	Thermo Scientific	Cat# AB-0796
Plastic screw thread autosampler vials	Thermo Scientific	Cat# C4000-12
Direct Detect assay-free cards	Merck Millipore	Cat# DDAC00010-GR

All reagents should be of Mass Spectrometry-grade quality. Single-use is recommended to avoid contamination.

## Materials and equipment

**CRITICAL:** Ensure adequate use of personal protection equipment (lab coat, gloves, facial mask, safety glasses) when handling the reagents listed below.

**Table:** Tris-lysis buffer (freshly prepared)

Reagent	Final concentration	Stock concentration	Add to 10 mL
Tris pH 8.0	10 mM	1 M	0.1 mL
Sodium Chloride (NaCl)	150 mM	5 M	0.3 mL
Triton X-100	1%		0.1 mL
EDTA	25 mM	0.5 M	0.5 mL
LC-MS grade water	-	-	9 mL
EDTA-free Roche complete protease inhibitor cocktail	-	-	1 tablet
**Total**	**-**	**-**	**10 mL**

This solution should be prepared freshly and kept on ice until use. Add 1 tablet of EDTA-free protease inhibitor cocktail and vortex to dissolve right before use.

**Table:** Alkaline detergent buffer (freshly prepared)

Reagent	Final concentration	Stock concentration	Add to 10 mL
Triton X-100	0.5%	-	0.05 mL
Phosphate-Buffered Saline (PBS)	0.1 M	-	9.9 mL
Ammonium hydroxide (NH_4_OH)	20 mM	5 M	0.04 mL
**Total**	**-**	**-**	**10 mL**

This solution should be prepared freshly and kept on ice until use. Add 0.04 mL of 5 M NH_4_OH right before using.

**CRITICAL:** Handle the NH_4_OH inside a fume hood.

• 100 mM dithiothreitol (DTT) solution: add 15.4 mg of DTT to 1 mL of LC-MS grade water.

This solution should be prepared freshly and kept on ice until use.

• 100 mM iodoacetamide (IAM) solution: add 18.5 mg of IAA to 1 mL of LC-MS grade water.

This solution should be prepared freshly and kept on ice until use, protected from light.

• OLIGO beads in acetonitrile (ACN): suspend the OLIGO R3 Reversed beads in 50% aqueous ACN.

Let the beads settle down before using and pipette from the bottom. The bead solution can be kept at 20°C in a sealed glass bottle for several weeks.

• Prepare a 1 M triethylammonium bicarbonate (TEAB) stock solution and adjust pH to 8.5 with phosphoric acid.

This solution can be frozen at −20°C and stored for several weeks.

**Table:** 2× TEAB/SDS lysis buffer∗

Reagent	Final concentration	Stock concentration	Add to 10 mL
Triethylammonium bicarbonate (TEAB), pH 8.5	100 mM	1 M	1 mL
Sodium dodecyl sulfate (SDS)	10%	-	1 g
LC-MS grade water	-	-	9 mL
**Total**	**-**	**-**	**10 mL**

Stable at 20°C for long-term storage (several weeks). Adjust to pH 7.5. Use LC-MS grade water to dilute to 1×.

• MTBE/MetOH solution∗: mix 7 parts of methyl-tert butyl ether (MTEB) with 3 parts of methanol (MetOH) (v/v).

This solution should be prepared freshly.

• Protein binding/washing buffer∗: mix 1 mL of 1 M TEAB stock solution with 9 mL of MetOH. Adjust pH to 7.1.

Final concentration: 100 mM TEAB, 90% MetOH. Store at 20°C for 2–3 weeks.

• Digestion buffer∗: mix 0.75 mL of the 1 M TEAB stock solution with 14.25 mL of LC-MS grade water. Adjust pH to 7.5.

Final concentration: 50 mM TEAB. Store at 20°C for 2–3 weeks.

• 12% phosphoric acid: for 10 mL, mix 1.4 mL of phosphoric acid (85%) with 8.6 mL of LC-MS grade water.

Store at 20°C in a glass bottle, for 2–3 weeks.

• 0.1% formic acid in 30% acetonitrile (ACN): for 15 mL, add 4.5 mL of ACN and 15 μL of formic acid to 10.5 mL of LC-MS grade water.

Store at 20°C in a glass bottle, for 2–3 weeks.

• 50% ACN: for 15 mL, mix 7.5 mL of ACN and 7.5 mL of LC-MS grade water.

Store at 20°C in a glass bottle, for 2–3 weeks.

∗Solutions prepared according to the ProtiFi S-Trap micro spin column digestion protocol (available at: https://protifi.com/pages/protocols/) and a protocol developed by Ronan O’cualain (available at: https://www.protocols.io/view/s-trap-column-digestion-protocol-protifi-of-protei-yxmvmn6q9g3p/v1/).

## Step-by-step method details

### Sample enrichment for extracellular matrix proteins

**Timing: 4–5 h (for step 1)**

**Timing: 30–60 min (for step 2) (may vary according to the number of samples and sonication protocol)**

**Timing: 1 h (for step 3)**

**Timing: 30–60 min (for step 4) (may vary according to the number of samples)**

For label-free quantitative discovery proteomics, we employ a chemical fractionation strategy^12^ to enrich fetal mouse kidneys and kidney organoids for the detection of extracellular matrix proteins. Although we describe here an application for mammalian kidney systems, this protocol has also been tested with different tissue types (e.g., skin, brain meninges, lungs, and heart) and organisms (e.g., Drosophila, *C. elegans*). For highly fibrous and/or keratinized tissues, additional steps may be necessary to ensure full solubilization of fractionation pellets.

**CRITICAL:** Ensure adequate use of personal protection equipment (lab coat, gloves, facial mask, safety glasses) throughout sample fractionation and following processing for mass spectrometry.

1. Day 1: Extracellular matrix enrichment through chemical fractionation and sonication.
  ***Note:*** Your samples were stored at −80°C, in sample tubes.
  a. Thaw kidney organoids and fetal mouse kidney samples and wash briefly with ice-cold PBS.
    **CRITICAL:** Samples must be kept on ice all the time to minimize protein degradation.
    ***Note:*** In this study, at least 3 kidney organoids per time point were collected and pooled whilst 6 mouse fetal kidney samples (2 kidneys per fetus, approximately 8 mg of tissue) were processed individually.
  b. Place the samples in clean Petri dishes and cut the samples into small pieces (roughly 1 mm^3^) using a sterile blade.
    ***Note:*** For adult kidney samples, remove the kidney capsule and pelvis prior to tissue homogenization as these compartments are fibrous and may interfere with the efficiency of the enrichment strategy.
    **CRITICAL:** Discard used blades in a biohazard sharps container.
  c. Add 0.5 mL–1 mL of ice-cold PBS to the tissue homogenate to aid in collecting and transferring it to 1.5 mL sample tubes.
  d. Incubate the samples for 5 min on a rotator to remove residual blood.
  e. Centrifuge at 10,000 × *g* for 1 min at 4°C and discard the PBS. Repeat this step at least once until all PBS has been removed.
  f. Add freshly prepared Tris-lysis buffer to samples tubes.
    i. For kidney organoids, add 50 μL of Tris buffer.
    ii. For mouse fetal kidneys, add 100 μL–200 μL of Tris buffer.
      ***Note:*** Use the smallest possible volume of Tris lysis buffer - high volumes might be difficult to handle when processing the samples for later trypsin digestion. Furthermore, very dilute samples will yield poor protein coverage in the proteomic analysis. We recommend a 5:1 (v/w) ratio for buffer and tissue, respectively. For approximately 3 mg–5 mg of tissue, 200 μL of lysis buffer might be enough for processing.
  g. Mechanically disrupt samples through repetitive pipetting using 200 μL pipette tips. Remember to keep the bottom of the tube on ice.
    ***Note:*** Consider using a syringe equipped with an 18- or 21-gauge needle to shear the partial homogenate by passing it through the needle, however, be careful with sample loss when using needles compared to pipette tips.
    **CRITICAL:** Discard used needles in a biohazard sharps container.
  h. Incubate the homogenate (Figure 1B) under gentle rotation (10 RPM) at 4°C to allow cell lysis and extraction of soluble proteins.
    ***Note:*** Incubation may vary from 30 min for cell-based samples to 1 h for tissue samples.
  i. Centrifuge the sample tubes at 14,000 × *g* for 10 min at 4°C.
  j. Collect the supernatant and transfer it to new sample tubes and label it as **Fraction 1 –** This is your first sample fraction that is enriched for soluble cellular proteins.
    **CRITICAL:** Be careful not to disturb the pellet while collecting the supernatant.
    ***Note:*** Tubes containing **Fraction 1** should be kept on ice while the fractionation is carried out or stored at −80°C until further processing.
  k. Resuspend the pellet in a freshly prepared alkaline detergent buffer by repetitive pipetting.
    ***Note:*** As for the Tris-lysis buffer, we recommend using the smallest possible alkaline buffer.
    **CRITICAL:** If the pellet is not fully resuspended, use a syringe equipped with an 18-gauge needle to shear by passing the partial pellet through the needle.
  l. Incubate under gentle rotation at 4°C for 30 min to 1 h to allow solubilization and disruption of cell-matrix interactions.
    **CRITICAL:** Incubate the samples in the alkaline buffer for no longer than 1 h as the NH_4_OH will damage proteins.
  m. Centrifuge the sample tubes at 14,000 × *g* for 10 min at 4°C.
  n. Collect the supernatant and transfer it to new sample tubes and label it as **Fraction 2** – This second sample fraction is less concentrated than **Fraction 1** and contains cell surface and transmembrane proteins.
    **CRITICAL:** Be careful not to disturb the pellet while collecting the supernatant.
    ***Note:*** Tubes containing **Fraction 2** should be kept on ice while the fractionation is carried out or stored at −80°C until further processing.
    ***Note:* Fraction 1** and **Fraction 2** may be combined in a 1:2 (v/v) proportion as a **Cellular Fraction** or processed separately.
  o. Resuspend the remaining pellet in 50 μL of 1× TEAB/SDS lysis buffer by repetitive pipetting, and label it as **Fraction 3** - this is the enriched **ECM fraction.** It will be a visible pellet in a range of around 50 μL.
    ***Note:*** At this stage, the pellet consists of a thick, viscous, transparent mixture of highly insoluble proteins, which may be difficult to resuspend completely into solution through manual homogenization. Proceeding to sonication will aid with dissolving the pellet.
    ***Note:*** Collect a desirable volume of **Fraction 1** and **Fraction 2**, or **Cellular Fraction**, and transfer to new sample tubes, and then add the same volume of 2× TEAB/SDS lysis buffer. The final concentration of SDS will be 5%, which will ensure protein denaturation and inactivation of undesirable protease digestion.
2. Day 1: Sample sonication.
  a. Place 50 μL of **ECM fraction** into Covaris AFA glass tubes.
    ***Note:*** The user can opt to add 5 mM of dithiothreitol (DTT) to the samples at this stage to improve disulfide reduction later after the sonication step.
  b. Sonicate the **ECM sample fraction** using a Covaris LE220+ operated through the SonoLab software. Optimize and set the sonication parameters according to sample type. For example:
    i. For kidney organoids, set duration = 30 s/sample, peak power = 500 W, duty factor = 20%, 50 cycles/burst, average power = 100 W.
    ii. For fetal mouse kidneys, set duration = 100 s/sample, peak power = 500 W, duty factor = 40%, 200 cycles/burst, average power = 200 W.
    iii. For ∗laser-captured or sieved glomeruli, set duration = 60 s/sample, peak power = 500 W, duty factor = 40%, 50 cycles/burst, average power = 200 W.
      ***Note:*** Other sonication approaches might also be considered and optimized to yield full disruption of the pellet without physically damaging proteins.
      ***Note:*** ∗In our study, the laser-captured fetal mouse glomeruli were not processed for matrix enrichment but resuspended in 1× TEAB/SDS lysis buffer and sheared by sonication.
  c. Place the sonicated **ECM sample fractions** on ice.
    ***Note:*** You may store the sample fractions in 1× TEAB/SDS lysis buffer at −80°C at this stage or proceed to the protein reduction and alkylation.
3. Day 1: Reduction and alkylation.
  a. Place 50 μL or 100 μL of sample fraction into clean sample tubes.
  b. Add 2.5 μL or 5 μL of 100 mM DTT stock solution to 50 μL or 100 μL of sample fraction, respectively, to a final concentration of 5 mM of DTT, and incubate for 10 min at 60°C using a heating block to reduce proteins.
  c. Let the samples cool down to 20°C then add 7.5 μL or 15 μL of 100 mM IAM stock solution to 50 μL or 100 μL of sample fraction, respectively, to a final concentration of 15 mM of IAM, and incubate for 30 min at room temperature, in the dark, to alkylate proteins.
  d. Add the same amount of DTT as previously to quench the residual alkylation reaction, and vortex mix briefly.
4. Day 1: Protein quantitation.
  a. To measure protein concentration, use Millipore Direct Detect Assay-free Cards and place 2 μL of (reduced, alkylated) protein lysate in the center of corresponding membrane positions sized spots (and 2 μL of blank, i.e., 1× TEAB/SDS lysis buffer, in the membrane designated as the blank position).
  b. Insert the card into a Millipore Direct Detect Spectrometer and read the corresponding protein concentration for each sample.

***Note:*** This system requires only 2 μL per analysis to provide accurate measurement of protein lysates. If your sample is highly concentrated, consider diluting it in 1× TEAB/SDS lysis buffer added with 5 mM DTT and 15 mM IAM. Alternatively, other protein quantification approaches such as colorimetric protein assays (Bradford, BCA) can be considered.

### Sample processing for label-free mass spectrometry-based proteomics

**Timing: 2 h (for step 5)**

**Timing: 16 h (for step 6)**

**Timing: 1 h (for step 7) (may vary according to the number of samples)**

**Timing: 2–4 h (for step 8)**

This section will detail the procedures for protein clean-up using ProtiFi S-Trap Spin Columns and subsequent protein digestion to peptides with trypsin. At this stage, the sample fractions consist of reduced, alkylated protein lysates in 50 mM TEAB (pH 7.5) containing 5% SDS. If the samples have been stored at −20°C, allow enough time for the samples to thaw at room temperature.

***Note:*** The steps described next were adapted and optimized from a ProtiFi S-Trap micro spin column digestion protocol (available at: https://files.protifi.com/protocols/s-trap-micro-kit-2-1.pdf ) and a protocol developed by Ronan O’cualain (available at: https://www.protocols.io/view/s-trap-column-digestion-protocol-protifi-of-protei-yxmvmn6q9g3p/v1/).

5. Day 2: Sample preparation for trypsin digestion in S-Trap Spin Columns.
  ***Note:*** Before starting, we recommend processing 25 μL or 50 μL of sample fractions to facilitate calculations for sample washing, cleaning, and digestion.
  ***Note:*** The ProtiFi S-Trap micro spin columns are equipped with derivatized silica that traps undigested proteins within its submicron pores, which allows washes to fully remove contaminants and detergents. The micro spin columns are suitable for samples with 1 μg–100 μg of proteins as per the manufacturer’s recommendations.
  ***Note:*** For the following steps, we will consider a sample volume of 50 μL, but adjustments can be made to other volumes accordingly.
  a. 50 μg of protein is needed as starting material (the volume of sample fraction required can be determined based on previous protein concentration measurements).
    ***Note:*** If not 50 μL of protein lysates, make it up to this volume with 1× TEAB/SDS lysis buffer.
  b. Add 5 μL of 12% (v/v) phosphoric acid (final concentration will be 1.2% phosphoric acid) and vortex mix briefly.
    ***Note:*** This step acidifies the lysate to pH < 1.0, which is critical to denature the proteins and aid the interaction with the resin within the spin columns.
  c. Add 330 μL of protein binding/washing buffer and vortex mix briefly.
  d. Place the spin columns on top of 2 mL collecting tubes, and add the acidified, methanolic protein lysates into the columns.
  e. Centrifuge at 4,000 × *g* for 2 min.
    **CRITICAL:** Because the spin columns fit approximately 200 μL, sequential loading, and centrifugation rounds will be necessary if your sample volume is > 200 μL until all the lysate has passed through the silica.
    ***Note:*** Do not let the liquid that passes through the spin column come in contact with the protein-trapping silica within the column.
  f. Wash the trapped proteins with 150 μL of MTBE/MetOH by simply adding the solution and centrifuging at 4,000 × *g* for 2 min and discarding the flow-through.
    ***Note:*** This wash with MTBE/MetOH will remove lipids that are not removed using methanol only.
  g. Wash with 150 μL of protein binding/washing buffer by simply adding the buffer followed by centrifugation at 4,000 × *g* for 2 min, and discard the flow-through. Repeat this wash at least three times.
    ***Note:*** Protein denaturation through incubation with protein solvent (SDS), acidification, and multiple exposure to high concentrations of methanol will eliminate any undesired protease activity, hence reducing proteolysis significantly, and maximizing the efficiency of trypsin digestion next.
6. Day 2: Trypsin digestion.
  a. Move the spin columns to clean 1.5 mL collecting tubes.
  b. Prepare 20 μg of Sequencing Grade Modified Trypsin (Promega) in 200 μL of digestion buffer.
    ***Note:*** This volume will be enough for 20 reactions (consider using no less than 1 μg of the protease of choice per 10 μg of protein/spin column).
  c. Add 20 μL of trypsin into the spin columns using a gel loading tip.
    **CRITICAL:** When adding the trypsin solution, do not touch the trapping silica to avoid contamination. Do not leave air bubbles between the trypsin solution and the trapping silica – the S-Trap binding silica is highly hydrophilic and will absorb the trypsin solution.
  d. Cap the spin columns to limit the evaporative loss and place the columns/collecting tubes on a heating block and incubate at 37°C for 12 h.
    ***Note:*** Alternatively, incubate for 1 h at 47°C. Do not shake or move the columns during incubation. Note that some dripping may occur during incubation, but this is not of concern.
7. Day 3: Peptide elution.
  After the incubation finishes, proteins will have been fully (or partially) digested to tryptic peptides. The next steps will recover the hydrophobic peptides from S-Trap trapping silica. From now on, we will keep and combine each elution after each centrifugation round to yield the peptide samples.
  a. Remove the spin column and collecting tubes very carefully from the heating block and allow a few minutes for them to cool down to room temperature.
  b. Add 65 μL of digestion buffer to the spin columns and centrifuge at 4,000 × *g* for 2 min and keep the flow-through.
  c. Add 65 μL of 0.1% aqueous formic acid and centrifuge at 4,000 × *g* for 2 min. Combine the flow-through with the first elution.
  d. Add 30 μL of 0.1% formic acid in 30% ACN and centrifuge at 4,000 × *g* for 2 min. Combine the flow-through with the previous elution.
    ***Note:*** The final elution volume is approximately 180 μL, and the final concentration of acetonitrile will be around 5% (v/v).
  e. The peptide samples are in an acidic mixture of organic solvents and salts. Proceed to desalt or store the elution at 4°C for 12 h.
8. Day 3: Peptide desalting, cleaning, and drying.
  a. Prepare the OLIGO R3 beads in 50% ACN in a glass vial and let them settle into the bottle of the vial.
  b. Add 10 μL of the settled OLIGO R3 beads into the appropriate number of wells of a Corning FiltrEX 96-well filter plate equipped on top of a 96-well collecting plastic plate.
  c. Wash the beads with 200 μL of 50% ACN.
  d. Centrifuge the plate at 200 × *g* for 1 min to remove the liquid.
    ***Note:*** Discard the liquid containing ACN to an adequate non-chlorinated solvent waste container.
  e. Wash the beads with 200 μL of 0.1% formic acid and centrifuge at 200 × *g* for 1 min to remove the liquid. Repeat this wash once.
  f. Add the peptide samples to the wells containing the beads and incubate on a plate mixer for 5 min at 300 RPM without heating.
  g. Centrifuge at 200 × *g* for 1 min.
    ***Note:*** Each well fits approximately 200 μL of liquid, hence, repeat the last two steps if there is an additional sample left to process.
  h. Add 200 μL of 0.1% formic acid to the wells containing the beads and peptides and incubate on a plate mixer for 2 min at 300 RPM without heating.
  i. Centrifuge at 200 × *g* for 1 min and discard the flow-through.
  j. Repeat the wash once.
  k. Replace the 96-well collecting plate with a new plate with unused, clean wells, to elute the peptides.
  l. Add 50 μL of 0.1% formic acid in 30% ACN to the wells containing the beads and peptides and incubate on a plate mixer for 2 min at 300 RPM without heating.
  m. Centrifuge at 200 × *g* for 1 min and keep the flow-through.
  n. Repeat the previous wash, incubation, and centrifugation.
  o. Combine the flow-through with the first elution – these are your peptide samples.
  p. Transfer the peptide samples to MS sample vials and label them accordingly.
    ***Note:*** The peptide sample volume is approximately 100 μL. We recommend preparing pooled samples for quality control check and to improve the coverage for protein identification in the proteomic runs: for every 10 samples, take 9 μL from each sample and add to another MS vial labeled “pool” – you now will have 10 samples with 91 μL of sample and a pooled sample of 90 μL.
  q. Completely dry the peptide samples using vacuum centrifugation.
    ***Note:*** Dried peptides are stable and can be kept at refrigerating temperature.

### Sample analysis and data acquisition with mass spectrometry

**Timing: 1–2 days (for step 9)**

In our study, the sample processing immediately prior to the analysis with high-resolution mass spectrometry was performed by the staff of the Biological Mass Spectrometry Facility (University of Manchester). Here we will describe the parameters used specifically for our study but can be adapted to other studies and/or instruments, accordingly.

9. Day 4: Sample analysis by liquid chromatography-tandem mass spectrometry (LC-MS/MS)
  a. Reconstitute the dry peptides in 12 μL of 0.1% formic acid in 3% ACN.
  b. Use the pooled samples for instrumental quality assessment and sample quality check.
  c. Calibrate the instrument as per requirement and prepare the setup for LC-MS/MS.
    ***Note:*** For our study, we used a Thermo Scientific Q Exactive HF hybrid quadrupole-Orbitrap mass spectrometer equipped with a Dionex Corporation UltiMate 3000 Rapid Separation LC nano-system.
  d. Peptide separation through liquid chromatography is carried out with an UltiMate 3000 RSLCnano System, using a CSH C18 analytical column operated at 300 nL/min flow rate.
    i. Mobile phase A is 0.1% formic acid in LC-MS water, and mobile phase B is 0.1% formic acid in 80% ACN.
    ii. The column used is a 75 mm × 250 μm, 1.7 μm CSH C18 column (Waters).
  e. Transfer 1 μL of peptide sample to a 5 μL loop and load onto the column at a flow of 300 nL/min for 5 min at 5% B.
  f. Use the following gradient for peptide separation:
    i. 5% B to 7% B from 300 nL/min to 200 nL/min over 1 min.
    ii. 7% B to 18% B over 64 min.
    iii. 18% B to 27% B over 8 min.
    iv. 27% B to 60% B over 1 min.
  g. Wash the column at 60% B for 3 min before re-equilibration to 5% B in 1 min.
  h. At 85 min, increase the flow to 300 nL/min until the end of the run at 90 min.
  i. Use the Q Exactive HF hybrid operated in the HCD mode.
  j. Set up peptide selection for fragmentation automatically by data-dependent acquisition on a basis of the top 12 peptides with m/z ranging from 300 to 1750 Th, charge state of 2, 3, or 4, with dynamic exclusion set at 15 s.
  k. Set MS resolution to 120,000, with an AGC target of 3^6^, and a maximum fill time of 20 ms.
  l. Set MS2 resolution to 30,000, with an AGC target of 2^5^, maximum fill time of 45 ms, isolation window of 1.3 Th, and collision energy of 28.
    ***Note:*** Parameters on the Q Exactive HF hybrid should be adjusted to different durations of runs.
    ***Note:*** ECM fractions were run first, followed by cellular fractions after. Blanks were run in between fractions and time-point for the organoid analysis, to avoid carryover from one sample to another.

### Processing and bioinformatics analysis of the proteomics data

**Timing: 1 day (for step 10)**

**Timing: 1–2 days (for step 11)**

10. Day 5: Proteome Discoverer analysis of cellular and ECM sample fractions.
  We used the Thermo Scientific Proteome Discoverer software. The software has default qualitative workflows utilizing Sequest HT for peptide/protein identification, and quantitative workflow for label-free assays.
  ***Note:*** More details about the software workflows are available through the user guide (available at: https://assets.thermofisher.com/TFS-Assets/CMD/manuals/Man-XCALI-97808-Proteome-Discoverer-User-ManXCALI97808-EN.pdf.
  ***Note:*** Users can use other search engines and software like Andromeda and MaxQuant, Progenesis and Mascot, or use combined search engines integrated into Proteome Discoverer as we describe later.
  **CRITICAL:** The cellular and ECM fractions are biologically distinct, i.e., their protein backgrounds are not comparable, and therefore, they should be analyzed separately.
  a. Download and save the spectra data (.raw) to a local folder in your computer.
  b. Open Proteome Discoverer and create a new study.
  c. In the new study definition tab, add and assign adequate study factors corresponding to your assay conditions and replicates (this will impact the way the protein and peptide ratios are calculated by the software; the reader can refer to the software user guide for details about study factors for nested and non-nested assay designs).
    i. Add “Individual” factors to assign sample replicate.
    ii. Add “Categorical” factors to assign experimental group or treatment.
      ***Note:*** In our kidney time course study, we defined “Day 14”, “Day 18”, and “Day 25” as categorical factors.
  d. In the “Input Files” tab, upload the .raw files for the analysis.
  e. In the “Samples” tab, assign to each .raw file its respective category (group or treatment) and individual (sample replicate).
    ***Note:*** In the “Samples” tab, keep all files assigned as “sample” in the “Sample Type” column.
  f. Create a “New Analysis” to open the Analysis window and two new tabs: “Workflows” and “Grouping and Quantification”.
  g. Select the “Consensus step” in the “Analysis” window, and in the “Workflows” tab, open the “CWF_Comprehensive_Enhanced Annotation_LFQ_and_Precursor_Quan” workflow from the “ConsensusWF” local folder – this is a default workflow for LFQ-proteomics.
    ***Note:*** In the “Precursor Ions Quantifier” node, you can opt to change the scaling mode to none, and keep all the default settings for the rest of the workflow tree.
  h. Select the “Processing step” in the “Analysis” window, and in the “Workflows” tab, open the “PWF_QE_Precursor_Quan_and_LFQ_SequestHT_Percolator” workflow from the “ProcessingWF_Qexactive” local folder – this is a default workflow for assays analyzed with the Q Exactive HF hybrid mass spectrometer.
  i. “Select the “Protein FDR Validator” and check the default settings for target FDR (strict = 0.01, relaxed FDR = 0.05). Proteins that pass the strict threshold will be classified and high confidence match whilst those passing the relax (but not the strict) threshold will be classified and medium confidence matches. Low confidence matches should not be considered for further analyses.”
    ***Note:*** Proteome Discoverer provides several default workflows for different instruments and analyses, with default settings. Select the adequate workflow accordingly.
    ***Note:*** Users are encouraged to use the latest released versions of the target database and additional contaminant database of choice.
  j. In the “Spectrum File RC” and “SequestHT” nodes, set Protein database to Homo sapiens or Mus musculus (SwissProt and TrEMBL databases), Precursor Mass Tolerance = 10 ppm, Fragment Mass Tolerance = 0.02 Da, and Dynamic Modifications = Oxidation / +15.995 Da (K, M, P), and leave the other setting as default.
    **CRITICAL:** For this study, we used the SwissProt and TrEMBL databases (v.2018_01; OS = Mus musculus for mouse samples; OS = Homo sapiens for kidney organoids).
    ***Note:*** Adding oxidation of proline (P) and lysine (K) as dynamic modifications improve the quantification and identification of peptides derived from the collagenous domain of collagen molecules, as these present random hydroxyprolines and hydroxylysines residues at this domain region.^12^^,^^13^^,^^14^
    ***Note:*** By default, Proteome Discoverer uses Sequest HT but other search engine nodes are available and can be integrated into the workflow tree. For each extra search engine node, integrate a new “Percolator” node.
  k. From the “Input Files” tab, select the appropriate files, and drag and drop them into the “Processing step” box.
  l. In the “Grouping and Quantification” tab, select the appropriate study variables (i.e., groups or treatment), and for the bulk ratio generator, choose the appropriate group as a denominator for the calculation of protein ratios.
    ***Note:*** In our time course study, we calculated “Day 18/Day 14”, “Day 25/Day 14”, and “Day 25/Day 18” ratios.
11. Label-free quantification and identification of extracellular matrix and basement membrane proteins
  a. Export the protein group files from Proteome Discoverer to an Excel file (.xls or .xlsx) and use this for the downstream analysis.
  b. To identify extracellular matrix and basement membrane proteins, download specie-specific ECM and ECM-associated protein lists from the matrisome project^15^ (https://sites.google.com/uic.edu/matrisome/home) and basement membrane proteins from bmBASE^5^ (https://bmbase.manchester.ac.uk/), and cross-reference with your data using gene symbols and/or UniProt identifiers.
    ***Note:*** matrisomedb^16^ and bmBASEdb^5^ are online searchable databases that provide lists of individual matrix and basement membrane proteins, respectively, which are classified into different categories: collagens, adhesive glycoproteins, proteoglycans, regulators, matrix-affiliated proteins, secreted factors, cell surface interactors, and others.
  c. For the downstream analyses, consider only proteins assigned to a unique “protein group”, quantified and identified by > 1 unique peptide, with a high/medium confidence match score (high: FDR < 1%, medium FDR > 1% and < 5% at protein level; use the same for peptide matches), and observed at least twice in each experimental group/condition.
  d. To calculate enrichment level for extracellular matrix and basement membrane proteins, first sum the intensity values (abundance) of all proteins in each respective category, then divide by the total sum of protein intensity to obtain the relative abundance of these protein categories per sample.
    ***Note:*** Apply the same strategy to calculate the relative abundance of the different matrix and basement membrane protein categories.
  e. Calculate the median extracellular matrix and basement membrane protein abundance per group and perform statistical comparisons with an ANOVA test.
    ***Note:*** In our study, no missing value imputation was done, and the statistical analyses were carried out with Proteome Discoverer through its in-built two-way ANOVA test with *post hoc* Benjamini-Hochberg correction, with proteins with a p-value < 0.05 being considered as significant.
    ***Note:*** If preferred, protein intensities can be log2-transformed prior to the abovementioned calculations to facilitate interpretation and visualization of the data.
    ***Note:*** For pairwise comparisons, use a student’s t-test or Welch’s t-test, depending on group size, Gaussian distribution of the data, and variance disparity between groups.
  f. Calculate fold changes for individual proteins and consider only those passing the statistical test with significant p-values (i.e., < 0.05).
    ***Note:*** All box plots and bar plots for data visualization were obtained using the GraphPad Prism software. The Principal Component Analysis, Hierarchical Clustering, and heatmaps were obtained using RStudio and the ggplot2 package.^10^
12. Data integration.
  In this step, we reprocessed proteomic data from human glomeruli and kidney tubulointerstitium (PRIDE: PXD026002, PXD022219) with Proteome Discoverer and further compared with human kidney organoid and mouse fetal kidney data with Spearman’s rank correlation.
  a. To calculate the correlation coefficient and generate correlation plots, perform analyses for the cellular and ECM fractions separately, using the ComplexHeatmap package^11^ for R.
13. Gene ontology.
  a. Prepare a list of differentially expressed proteins (for example 2-times fold-change, p-value < 0.05 or FDR < 0.05; or up- and/or down-regulated) for the gene ontology (GO) analysis.
  b. Prepare a second list of all proteins detected in your system (kidney organoids, mouse kidneys) for use as the protein background for GO.
    ***Note:*** In our study, we used DAVID Bioinformatics Resources^17^ or GO. Readers can find a detailed tutorial at https://david.ncifcrf.gov/helps/tutorial.pdf.
  c. Go to https://david.ncifcrf.gov/and start the analysis.
  d. Step 1: upload the protein background, the list of proteins differentially expressed, and select the adequate identifier (e.g., “OFFICIAL_GENE_SYMBOL” if using gene names).
    ***Note:*** When using gene names, indicate the adequate species for the analysis (e.g., *Homo sapiens* for the kidney organoids, *Mus musculus* for the mouse kidney).
  e. Submit the lists.
  f. Step 2: for the functional annotation tools, select the desired levels for GO annotation.
    ***Note:*** In our study, we searched for Gene_Ontology “DIRECT” terms, and “FAT” for more specific terms.
  g. Click on “Functional Annotation Chart” to generate a GO report with GO terms and adjusted statistical significance (Benjamini-Hochberg).
    ***Note:*** For mapping to signaling pathways, users can use Reactome^18^ (https://www.reactome.org/) as detailed here https://www.reactome.org/userguide/analysis/.
14. Interactome analysis.
  ***Note:*** In our study, we used the STRING database^19^ (https://www.string-db.org/) and Cytoscape^8^ to generate and customize protein interactomes. The reader can find detailed STRING tutorials at https://www.string-db.org/cgi/help/.
  a. Go to STRING and search interactions for a list of multiple protein candidates.
  b. Set a minimum interaction score of > 70% to obtain high-confidence interactions.
  c. Export the data as a short tabular text output to upload to Cytoscape.
  d. Generate and customize a protein interactome using the data from STRING.
    ***Note:*** Users can use the stringApp plugin^20^ (https://apps.cytoscape.org/apps/stringapp) to generate protein interactomes directly with Cytoscape. Network topology parameters can be computed and analyzed with the NetworkAnalyzer plugin^21^ (https://apps.cytoscape.org/apps/networkanalyzer).

## Expected outcomes

**Figure 2:**
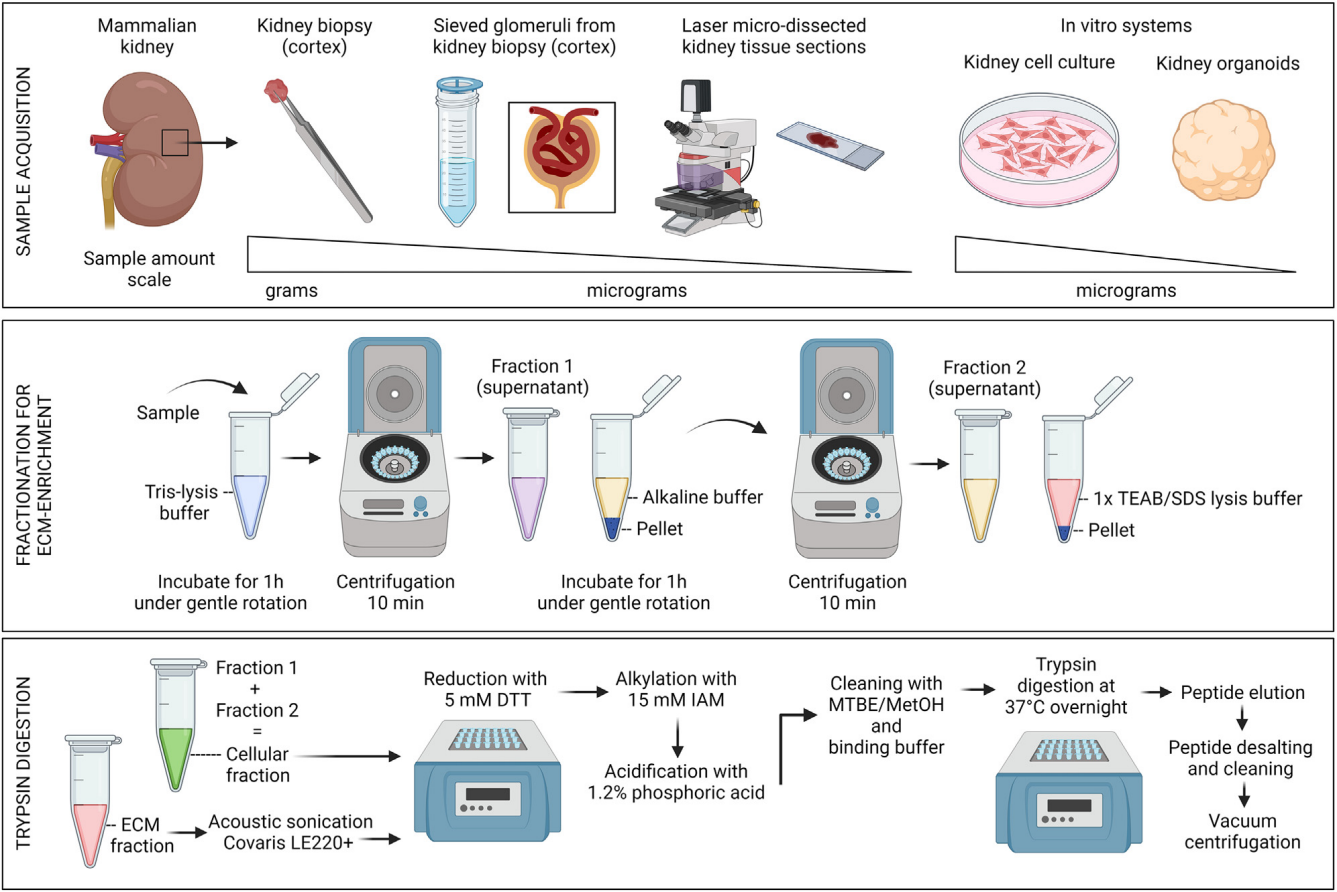
Sample acquisition and workflow for sample fractionation and processing for trypsin digestion for mass spectrometry analysis Created with BioRender.com with publication license agreement number: HD25XU5O15.

**Figure 3:**
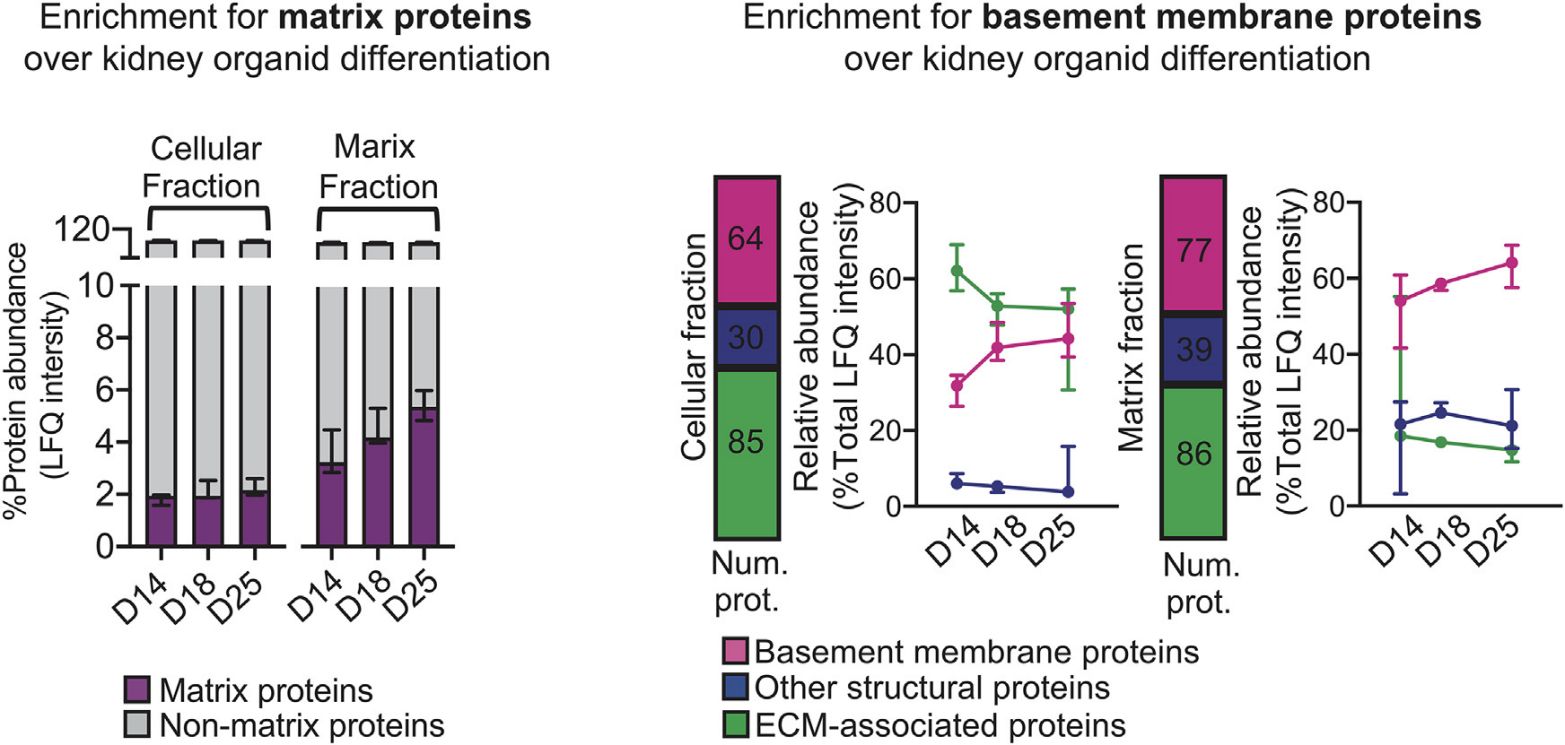
The expected level of enrichment for matrix and basement membrane proteins during human kidney organoid differentiation The plot on the left shows the relative abundance of matrix proteins identified and quantified in the cellular and ECM fractions of kidney organoids at days 14, 18, and 25 of differentiation, through our fractionation strategy; on the right, the plot indicates the crescent level of basement proteins quantified in the kidney time course study. Pooled data are presented as median, and error bars indicate the 95% confidence interval for the median. Adapted from Morais, Tian et al. under a CC BY 4.0 Attribution license [creativecommons.org] with permission.

To date, several ECM enrichment and purification strategies and processing approaches have been developed and successfully applied by other groups to unravel the molecular complexity and structure of ECM in different biological conditions.^14^^,^^22^^,^^23^^,^^24^^,^^25^^,^^26^^,^^27^^,^^28^^,^^29^^,^^30^ Our strategy (Figure 2) mostly relies of differential solubility of ECM and ECM-associated proteins, and with it we have defined the global composition of basement membranes in cell-based and animal tissue systems, and specifically elucidated molecular changes throughout organoid differentiation (Figure 3). This strategy is also expected to reveal the composition of interstitial matrices and changes in diverse contexts, such as development and disease. By fractionating the samples used in our study, we could identify and quantify over 6,600 proteins in kidney organoids, and over 5,000 in fetal mouse kidneys.^1^ The depth of coverage for identification and quantification of matrix and basement proteins with our enrichment strategy is expected to be higher than for assays using whole tissue lysates (Figure 3), but can also depend on several factors mentioned in this protocol, such as sample amount for fractionation, the inclusion of post-translational modifications for protein identification, the efficiency of trypsin digestion, the performance of the mass spectrometer, and the use of data-dependent acquisition (DDA) versus data-independent acquisition (DIA) modes, etc.

## Limitations

An important limitation in our approach is the absence of effective tissue compartmentalization during the processing of tissue fractions. However, this limitation can be addressed through the isolation of distinct tissue compartments. For instance, differential sieving or laser microdissection can be employed to separate kidney glomeruli and tubules. This isolation step may result in a substantial reduction in the initial quantity of tissue samples available, which in turn could affect the yield of fractionation and pose challenges in achieving a comprehensive proteome coverage. This is particularly applicable when dealing with low-abundance or insoluble matrix proteins, such as basement membrane collagens.

## Troubleshooting

### Problem 1

ECM-enriched pellet may be difficult to solubilize during the fractionation (step 1o).

### Potential solution

In this case, we recommend homogenizing the samples properly prior to the fractionation incubations, using a sterile blade and a syringe equipped with a gauge needle. Optimizing the sonication parameters may facilitate the dissolution of ECM-enriched pellet, and repeated rounds of sonication may be necessary. Furthermore, consecutive solubilization and pelleting down samples may be beneficial; however, this may increase the number of fractions, which will increase the cost for analysis.

### Problem 2

Incomplete trypsin digestion of protein lysate (step 6).

### Potential solution

The suggested trypsin-to-sample ratio of 1:10 should efficiently digest the recommended protein concentration. In case of incomplete digestion, it may be beneficial to conduct multiple rounds of trypsin digestion, either at 37°C for 12 h or at 47°C for 60 min. Alternatively, consider multi-enzyme digestion, such as using trypsin with LysC in conjunction with collagenase.^31^ It is important to note that opting for multi-enzyme digestion will have implications for the subsequent bioinformatics analysis, and this consideration should be factored into your experimental planning.

### Problem 3

Results show lower protein identification coverage (step 11).

### Potential solution

The success of the fractionation relies partially on having enough tissue samples. Pooling samples prior to the enrichment step may be considered to increase the amount of starting material to later improve the number of proteins identified in the proteomic analysis. The user should consider individual and batch-to-batch variations when interpreting the results. Alternative approaches to enhance ECM protein identification may include deglycosylation of tissue lysates with glycosidases.^32^ Data Independent Acquisition (DIA) might also be considered to increase the coverage for protein identifications, but the user should consider the caveats of data processing and analysis if using DIA data analysis tools.^33^^,^^34^^,^^35^^,^^36^

### Problem 4

Results show multiple entries for the same protein or protein group (step 11).

### Potential solution

This often happens due to multiple spectra/peptides that match the same protein or protein group. We recommend reviewing the protein sequence database used in the search. Use the most up-to-date version of the database and ensure that it only includes relevant/unique protein entries to minimize redundancy of the results. Ensure that you are filtering out low-confident peptides, and those that are not unique and confidently matched to specific proteins. Additionally, avoiding the use of databases that include multiple entries for different isoforms or protein fragments, such as TrEMBL Fasta files, can help resolve ambiguities in the results.

### Problem 5

Batch-to-batch and inter-batch variations for the kidney organoids (step 10f).

### Potential solution

Due to batch-to-batch variation of kidney organoid differentiation driven by differences in the rates of organoid maturation, we recommend processing organoids from the same batch to achieve comparable datasets. We also suggest the practice of pooling samples to reduce inter-batch variations. On the other hand, batch-to-batch variation could help identify robust signatures/differences.

## Resource availability

### Lead contact

Further information and requests for resources and reagents should be directed to and will be fulfilled by the lead contact, Rachel Lennon (Rachel.Lennon@manchester.ac.uk).

### Materials availability

This study did not generate any new unique reagents and/or materials.

### Data and code availability

The mass spectrometry proteomics data have been deposited to the ProteomeXchange Consortium via the PRIDE partner repository (Perez-Riverol et al., 2019) with the dataset identifiers: PXD025838, PXD025874, PXD025911, and PXD026002.
